# Long‐term outcomes after biliary diversion operation for pancreaticobiliary maljunction in adult patients

**DOI:** 10.1002/ags3.12239

**Published:** 2019-02-19

**Authors:** Takanori Aota, Shoji Kubo, Shigekazu Takemura, Shogo Tanaka, Ryosuke Amano, Kenjiro Kimura, Sadaaki Yamazoe, Hiroji Shinkawa, Go Ohira, Toshihiko Shibata, Masaki Horiike

**Affiliations:** ^1^ Department of Hepato‐Biliary‐Pancreatic Surgery Osaka City University Graduate School of Medicine Osaka Japan; ^2^ Department of Pediatric Surgery Osaka City University Graduate School of Medicine Osaka Japan

**Keywords:** biliary tract cancer, cholangitis, congenital biliary dilation, hepatolithiasis, pancreaticobiliary maljunction

## Abstract

**Aim:**

Pancreaticobiliary maljunction (PBM) with or without congenital biliary dilatation (CBD) is a risk factor for biliary tract cancer. We investigated long‐term outcomes after biliary diversion operation with special reference to types of CBD.

**Methods:**

Subjects comprised 40 adult patients who underwent biliary diversion operation for PBM without biliary tract cancer. Group A comprised 20 patients with type Ia or Ic CBD, or non‐dilated bile ducts, while group B comprised 20 patients with type IV‐A CBD. The clinical findings and postoperative outcomes were compared between groups.

**Results:**

Of 40 patients, nine patients suffered from repeated cholangitis and eight of these nine patients suffered from hepatolithiasis after biliary diversion operation. Biliary tract cancer or pancreatic cancer was detected in four patients at 3 years and 2 months to 24 years after the operation. In three of these four patients, the serum concentration of carbohydrate antigen 19‐9 increased before detection of carcinoma. One patient died of hepatic failure due to repeated cholangitis. The proportions of patients with repeated cholangitis, hepatolithiasis, and re‐operation, and patients who died of biliary tract cancer, pancreatic cancer, or hepatic failure, were significantly higher in group B than in group A. The survival rate was significantly worse in group B than in group A.

**Conclusions:**

Careful long‐term follow‐up with measurement of serum tumor markers is necessary after biliary diversion operation for PBM, especially in patients with type IV‐A CBD or repeated cholangitis.

## Abstract 2

This study showed that careful long‐term follow‐up with measurement of serum tumor markers is necessary after biliary diversion operation for PBM, especially in patients with type IV‐A CBD who were found to have significantly less favorable outcomes.

## 1 INTRODUCTION

Pancreaticobiliary maljunction (PBM) is a congenital malformation in which the pancreatic and bile ducts merge anatomically outside the duodenal wall, causing bile and pancreatic juice to flow into each other because the sphincter of Oddi cannot control the junction. In most patients, PBM is associated with congenital biliary dilatation (CBD). It is well known that PBM with or without CBD is a risk factor for biliary tract cancer.^1^, ^2^, ^3^, ^4^, ^5^, ^6^ The reflux of pancreatic juice into the biliary tract caused by PBM is thought to play a significant role in the development of biliary tract cancers, such as gallbladder cancer and cholangiocarcinoma in dilated bile ducts.^6^ Therefore, excision of the extrahepatic bile duct and cholecystectomy with hepaticojejunostomy using Roux‐en‐Y (the so‐called biliary diversion operation) is recommended to reduce the risk of biliary tract cancer.^5^ Late postoperative complications after the biliary diversion operation are known to include cholangitis and hepatolithiasis. In addition, it has been reported that biliary tract cancer can develop after the biliary diversion operation.^7^, ^8^, ^9^, ^10^ Previous studies have reported that the rates of these complications seem to be higher in patients with type IV‐A CBD compared to those with other types of CBD.^6^, ^9^, ^11^, ^12^ However, previous studies did not compare long‐term outcomes after the biliary diversion operation between patients with type IV‐A CBD and those with other types of CBD. In this study, we investigated the long‐term outcomes including biliary tract cancer, after the biliary diversion operation in adult patients, with a special reference to the types of CBD.

## 2 PATIENTS AND METHODS

### 2.1 Patients

The subjects in this study were 40 adult patients (eight men, 32 women) who underwent excision of the biliary diversion operation for PBM without biliary tract cancer (Table 1). For the purpose of this study, the biliary diversion operation was defined as excision of the total (dilated or non‐dilated) extrahepatic bile duct and cholecystectomy with hepaticojejunostomy using Roux‐en‐Y to prevent reflux of pancreatic juice into the biliary tract and reflux of bile into the pancreatic duct. PBM was diagnosed according to the diagnostic criteria for PBM by the Japanese Study Group on PBM.^13^ CBD was diagnosed according to the diagnostic criteria for CBD^14^ and the type of CBD was classified according to Todani's classification.^7^ Patient ages ranged from 20 to 73 years (median 45). Of the 40 patients, 38 had CBD and two had non‐dilated extrahepatic bile ducts. Of the 38 patients with CBD, the type of CBD was classified as type Ia in nine patients, type Ic in nine patients, and type IV‐A in 20 patients.

**Table 1:** Characteristic of patients who underwent excision of the extrahepatic bile duct for pancreaticobiliary maljunction according to the type of congenital biliary dilatation

Findings	Types		*P*
	Ia, Ic, no dilatation^a^	IV‐A	
	(n = 20)	(n = 20)	
Age	46 (20‐73)	44 (26‐67)	.880
Gender (male:female)	02:18	06:14	.235
Comorbidity			
Biliary stone	2	6	.235
Pancreatitis	1	1	>.999
Congenital diseases	1 (IDD)	1 (Annular pancreas)	>.999
I PMN	0	1	>.999
Previous operation			
Cholecystectomy (alone)	1	0	>.999
Choledochoduodenostomy	1	4	.342
Choledochojejunostomy	0	1	>.999
Liver resection^b^	1	0	>.999
With hepatectomy	1	4	.342

IDD, intraductal duodenal diverticulum; IPMN, intraductal papillary mucinous neoplasm.

a Type Ia (n = 9), Ic (n = 9), no dilatation (n = 2).

b Posterior segmentectomy of the liver without excision of extrahepatic bile duct.

Of the 40 patients, five had previously undergone choledocho‐duodenostomy (cyst‐duodenostomy), one had previously undergone choledocho‐jejunostomy (cyst‐jejunostomy), one had previously undergone cholecystectomy, and one had previously undergone posterior segmentectomy for hepatolithiasis. At the biliary diversion operation, seven patients had hepatolithiasis, and four of these seven had undergone choledocho‐duodenostomy or choledocho‐jejunostomy. Of the 40 patients, liver resection was also performed at the time of the biliary diversion operation in five patients.

One patient had a history of distal gastrectomy for gastric cancer. One patient was associated with intraluminal duodenal diverticulum^15^ and another patient was associated with annular pancreas.

### 2.2 Follow‐up

After the biliary diversion operation, patients were followed up at least once every few years for laboratory tests, including serum tumor markers such as carcinoembryonic antigen and carbohydrate antigen (CA) 19‐9 and diagnostic imaging such as computed tomography (CT), magnetic resonance imaging including magnetic resonance cholangiopancreatography, and ultrasonography. The long‐term outcomes including repeated cholangitis, hepatolithiasis, hepatic failure, biliary tract cancer, and pancreatic cancer, which are recognized as serious complications of this procedure, were retrospectively investigated.^5^, ^6^, ^7^, ^8^, ^9^, ^10^, ^16^, ^17^, ^18^ When oral or intravenous administration of antibiotics for cholangitis occurred twice or more per year, or when patients had hepatolithiasis caused by cholangitis, it was classified as repeated cholangitis. Fluctuation of serum amylase activity was also investigated, which was defined as serum amylase activities exceeding the reference range of 123 IU/L several times during the follow‐up period.

We divided the patients into two groups: group A comprised 20 patients who were classified as type Ia, Ic, or no dilation of the bile ducts, whereas group B comprised 20 patients who were classified as type IV‐A. The clinical findings and postoperative outcomes, including overall survival curves after the biliary diversion operation, were compared between the two groups. The median follow‐up period between the operation and death or end of this study (August 15, 2018) was 3319 days (range, 171‐12 716) in group A and 3714 days (range, 140‐9678 days) in group B.

This study was conducted following the guidelines of the Declaration of Helsinki and was approved by the ethics committee of Osaka City University (No. 4013).

### 2.3 Statistics

Continuous variables were compared between groups using Student's *t* test or Mann‐Whitney *U* test. Categorical data were compared using Fisher's exact test. We used the Kaplan‐Meier method to calculate the overall survival rates and differences between the two groups, which were evaluated using the log‐rank test. A *P* value of <.05 was considered statistically significant. All statistical analyses were performed using SPSS^®^ v.22.0 software (IBM Corp., Armonk, NY, USA).

## 3 RESULTS

### 3.1 Outcomes after the biliary diversion operation in all patients

**Figure 1:**
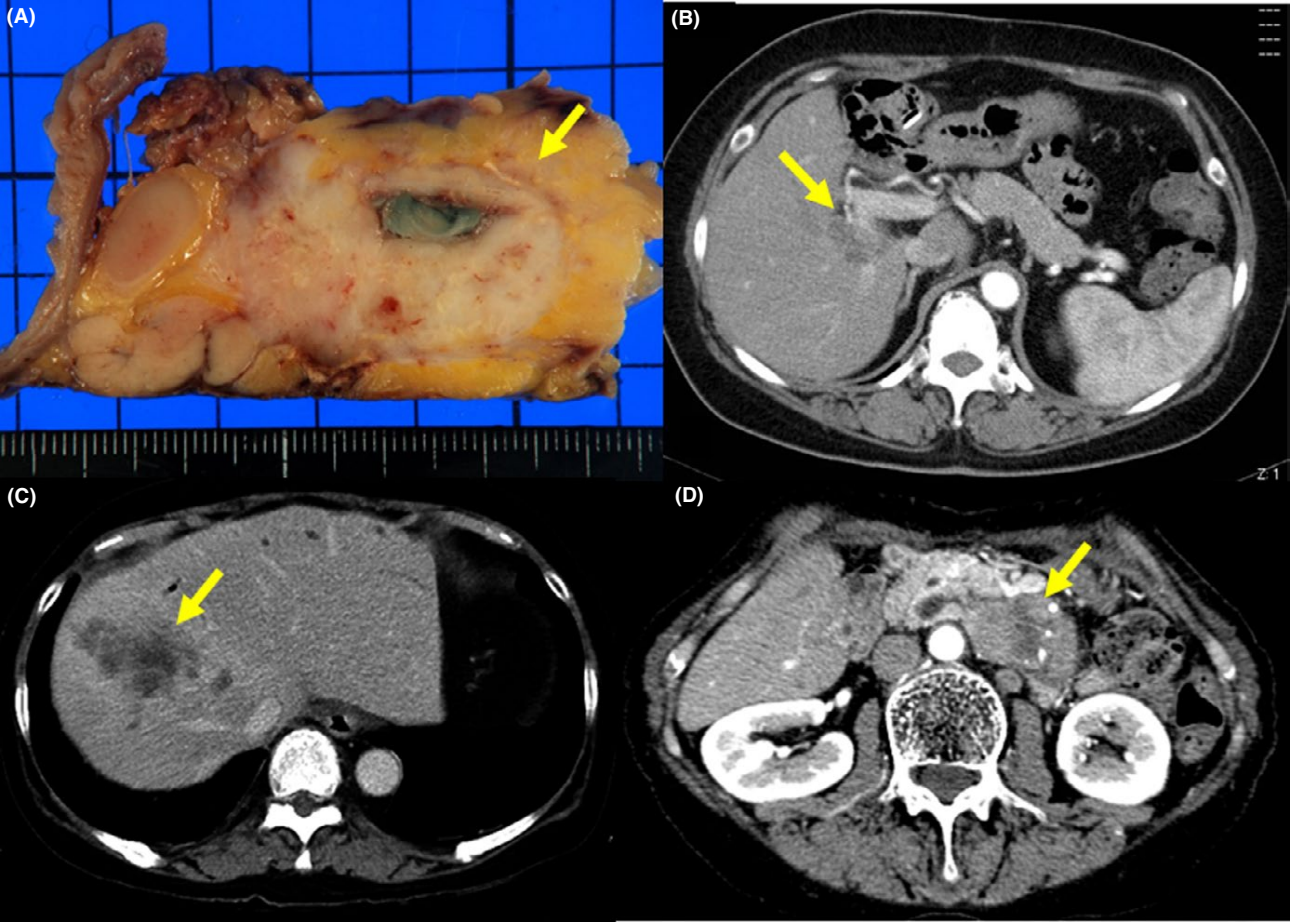
Cholangiocarcinoma and pancreatic cancer detected after the biliary diversion operation. A, Cholangiocarcinoma (arrow) is present around the residual bile duct in the pancreas. B, Space‐occupying lesion (hilar cholangiocarcinoma) is present (arrow). C, Space‐occupying lesion (intrahepatic cholangiocarcinoma) is present (arrow). D, Space‐occupying lesion (pancreatic cancer) is present (arrow)

Of the 40 patients, repeated cholangitis occurred in nine patients and hepatolithiasis developed in eight of these nine patients after the biliary diversion operation. During the follow‐up periods, biliary tract cancer developed in three patients and pancreatic cancer developed in one patient (Table 2). In one patient (type IV‐A), cholangiocarcinoma originating from the residual bile duct in the pancreas (Figure 1A) was detected 24 years after the biliary diversion operation (at age 31) following previous choledocho (cyst)‐duodenostomy (at age 15). Although pancreaticoduodenectomy was performed for the cholangiocarcinoma, the patient died of the cancer recurrence. Hilar cholangiocarcinoma (Figure 1B) was detected 3 years and 2 months after the biliary diversion operation (at age 48) in one patient (type IV‐A). Although the patient received chemotherapy, the patient died of the cholangiocarcinoma. Intrahepatic cholangiocarcinoma (Figure 1C) was detected 4 years and 1 month after the biliary diversion operation (at age 67) in one patient (type IV‐A). The patient received conservative therapy, including transhepatic biliary drainage, because of the advanced stage but the patient died. In another patient (type IV‐A), pancreatic cancer with multiple liver metastases (Fig. 1D) was detected 5 years and 8 months after the biliary diversion operation (at age 44). In this patient, fluctuation in serum amylase activity (around 200 IU/L) was observed after the biliary diversion operation. Although the patient received chemotherapy, the patient died of pancreatic cancer. In three of four patients with cholangiocarcinoma or pancreatic cancer, the serum concentration of CA 19‐9 increased before the detection of cholangiocarcinoma or pancreatic cancer. In 36 patients without cholangiocarcinoma or pancreatic cancer, a transient increase in serum concentration of CA19‐9 was detected in two patients with cholangitis.

**Table 2:** Characteristics of patients with biliary or pancreatic cancer after excision of extrahepatic bile duct for pancreaticobiliary maljunction

Age/sex	Previous operation	Type of biliary dilatation	Duration between operation and cancer detection	Site of cancer	Treatment for cancer
31/M	Choledocho‐duodenostomy	IV‐A	24 y	Intrapancreatic BD	Pancreatoduodenectomy
48/F	None	IV‐A	3 y, 2 mo	Hilar BD	Chemotherapy
67/F	None	IV‐A	4 y, 1 mo	Intrahepatic BD	PTBD
44/F	None	IV‐A	5 y, 8 mo	Pancreas	Chemotherapy

BD, bile duct; PTBD, percutaneous transhepatic biliary drainage.

In one patient who had undergone choledocho (cyst)‐duodenostomy for type IV‐A at age three, a left lobectomy with excision of the extrahepatic bile duct and anastomosis between the right hepatic duct and jejunum was performed for repeated cholangitis 34 years after the first operation because the intrahepatic bile ducts were dilated mainly in the left lobe. During the operation, cholangiofiberscopy showed multiple membranous stenoses in the intrahepatic bile ducts and septal stenosis at the hepatic duct. We resected the stenoses as much as possible. Recurrent cholangitis with hepatolithiasis developed after the surgery and lithotomy by endoscopy was repeated for the hepatolithiasis. However, the serum concentration of total bilirubin increased 7 years after the operation and the patient died of liver failure 10 years 2 months after the biliary diversion operation.

Of 40 patients, re‐operation under laparotomy was performed in five patients. Pancreaticoduodenectomy was performed in one patient for cholangiocarcinoma, as described earler. Liver resection was performed in two patients for recurrent cholangitis and hepatolithiasis. Re‐anastomosis of the hepaticojejunostomy was performed in two patients for stenosis that caused recurrent cholangitis with or without hepatolithiasis.

### 3.2 Relationship between pre‐ and postoperative status and postoperative complications

In group A, only one patient with preoperative repeated cholangitis developed hepatolithiasis. In group B, six of eight patients with preoperative repeated cholangitis developed cholangiocarcinoma or hepatolithiasis postoperatively while three of 12 patients without such cholangitis developed these complications (*P* = 0.0648). Postoperative cholangiocarcinoma or hepatolithiasis developed in eight of eight patients with postoperative repeated cholangitis but only one of 12 patients without such cholangitis (*P* = .0001). Of the 40 total patients, cholangiocarcinoma or hepatolithiasis developed after the operation in nine of nine patients with postoperative repeated cholangitis but only one of 31 patients without cholangitis (*P* < .0001). Thus, the rate of cholangiocarcinoma or hepatolithiasis after the biliary diversion operation was significantly higher in patients with postoperative repeated cholangitis than in patients without such cholangitis, especially in group B.

Pancreatic cancer developed in one of three patients showing postoperative fluctuation in serum amylase activity, and none in 37 patients who did not show postoperative fluctuation in serum amylase activity (*P* = .0750).

### 3.3 Comparison of postoperative outcomes between groups

**Figure 2:**
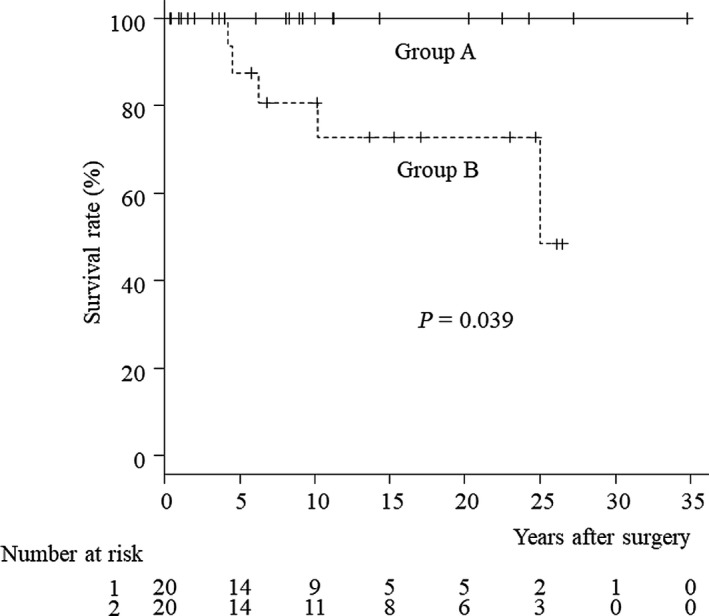
Survival rates after the biliary diversion operation according to the types of congenital biliary dilatation. Group A comprised 20 patients classified as type Ia, Ic, or no dilation of the bile ducts, while group B comprised 20 patients classified as type IV‐A

The proportions of patients with repeated cholangitis and hepatolithiasis were significantly higher in group B (type IV‐A) than in group A (other types; *P* = .0197 and *P* = .0436, respectively; Table 3). Although no patients in group A died during the follow‐up period, four patients in group B died of cholangiocarcinoma, pancreatic cancer, or hepatic failure. The proportion of deceased patients was significantly higher in group B than in group A (*P* = .0471). The proportion of patients who required re‐operation was significantly higher in group B than in group A (*P* = .0471). Figure 2 shows the survival curves after the operation in the two groups. The survival rate was significantly worse in group B than in group A (*P* = .039).

**Table 3:** Outcomes after excision of extrahepatic bile ducts according to the type of congenital biliary dilatation

Outcomes	Types		*P*
	Ia, Ic, no dilatation(n = 20)	IV‐A(n = 20)	
Repeated cholangitis	1	8	.0197
Hepatolithiasis	1	7	.0436
Death	0	5	.0471
Cholangiocarcinoma	0	3	
Pancreatic cancer	0	1	
Hepatic failure	0	1	
Re‐operation	0	5	.0471

## 4 DISCUSSION

Of the 40 patients in this study, nine suffered from repeated cholangitis and eight of these nine suffered from hepatolithiasis after the biliary diversion operation. Biliary tract cancer developed in three patients and pancreatic cancer developed in one patient after the operation. One patient died of hepatic failure caused by repeated cholangitis and hepatolithiasis. The rate of cholangiocarcinoma or hepatolithiasis after the biliary diversion operation was significantly higher in patients with postoperative repeated cholangitis than in patients without such cholangitis, especially in group B. As the result, the proportions of patients with repeated cholangitis or hepatolithiasis, patients who died of biliary tract cancer, pancreatic cancer, or hepatic failure, and patients who underwent re‐operation under laparotomy were significantly higher in group B (type IV‐A) than in group A (other types). The survival rate was significantly lower in group B than in group A.

In a Japanese nationwide survey, biliary tract cancer was observed in 215 (21.6%) of 997 adult patients with PBM and CHD and in 218 (42.4%) of 514 adult patients with PBM without biliary dilatation.^4^ Regarding the development of biliary tract cancer after the biliary diversion operation for PBM with or without CBD, Watanabe et al^8^ reported that the incidence was 0.7% (nine out of 1291 patients) in a review of the Japanese literature. Kobayashi et al^9^ reported that biliary tract cancer developed after the biliary diversion operation in three (5.4%) of 56 patients. Ohashi et al^10^ reported that cancer developed in four (4.3%) of 94 patients and that the sites of biliary tract cancer were intrahepatic (n = 2), hilar (n = 1), and intrapancreatic (n = 1). Ohashi et al. also reported that the cumulative incidence was 11.3% at 25 years. In this study, biliary tract cancer developed in three (7.5%) out of 40 patients after the biliary diversion operation, namely intrahepatic (n = 1), hilar (n = 1) and intrapancreatic (n = 1). Therefore, biliary tract cancer developed in approximately 10% of patients who underwent the biliary diversion operation. Watanabe et al^8^ reported that the interval between cyst excision (the biliary diversion operation) and the detection of the cancer after the biliary diversion operation was 1‐19 years (average, 9.0 ± 5.5 years). Kobayashi et al^9^ reported that cancer developed at 19 years and 6 months, 8 years and 8 months, and 2 years and 5 months after the biliary diversion operation. Ohashi et al^10^ reported that cancer developed at 13, 15, 23, and 32 years after the operation. In this study, biliary tract cancer was detected at 3 years and 2 months, 4 years and 1 month, and 24 years after the biliary diversion operation. Therefore, it is necessary to pay attention to cancer development for the long‐term even after the biliary diversion operation.

Kobayashi et al^9^ reported that CBD in all three patients with biliary tract cancer after the biliary diversion operation was classified as type IV‐A. Ohashi et al^10^ reported four patients with such biliary tract cancer, two of whom had type IV‐A CBD. In this study, all three patients with biliary tract cancer after the biliary diversion operation had type IV‐A CBD. Notably, the proportion of patients with repeated cholangitis with or without hepatolithiasis was significantly higher in group B (type IV‐A) than in group A. Furthermore, the rate of cholangiocarcinoma or hepatolithiasis after the biliary diversion operation was higher in patients with pre‐ or postoperative repeated cholangitis than in patients without cholangitis, especially in group B. Dilated intrahepatic bile ducts and ductal stenosis, such as membranous and septal stenosis,^6^, ^19^ and bilioenteric anastomosis can induce repeated cholangitis, which is responsible for the development of hepatolithiasis and biliary tract cancer. In this study, the serum concentration of CA 19‐9 increased gradually before the detection of biliary tract cancer or pancreatic cancer in three of four patients, although a transient increase in the serum concentration of CA 19‐9 was observed in two patients with cholangitis. We can conclude that careful long‐term follow‐up including measurement of serum tumor markers such as CA 19‐9 is important, even after the biliary diversion operation, especially in patients with type IV‐A CBD or repeated cholangitis.

As described earler, the proportions of patients with repeated cholangitis or hepatolithiasis in this study was significantly higher in group B than in group A. In addition, one patient who had undergone choledocho (cyst)‐duodenostomy (internal drainage operation) and underwent the biliary diversion operation 34 years after the first operation for the repeated cholangitis, died of hepatic failure due to secondary biliary cirrhosis caused by repeated cholangitis with hepatolithiasis. It has been reported that cyst‐enterostomy (internal drainage operation) enhances the risk of postoperative cholangitis and carcinogenesis.^20^ In addition, patients with type IV‐A CBD often have multiple and septal stenoses of the bile ducts.^6^, ^19^ Therefore, cyst‐enterostomy should be avoided. Furthermore, wide anastomosis of the hepaticojejunostomy and removal of stenoses is important in patients with type IV‐A CBD.^5^, ^19^, ^21^ From the viewpoint of risk of repeated cholangitis, hepatolithiasis, and cholangiocarcinoma, liver resection should be considered for patients with type IV‐A CBD when intrahepatic bile duct dilatation is present in one or two segments of the liver, although some patients still may have serious problems such as repeated cholangitis and hepatolithiasis.^11^

In this study, cholangiocarcinoma developed in the intrapancreatic bile duct in one patient. Therefore, the intrapancreatic bile duct should be dissected at the level immediately above the pancreaticobiliary junction, so as to leave as little intrapancreatic bile duct as possible. To resect the intrapancreatic bile duct as much as possible, intraoperative cholangiography using a metal clip or biliary endoscopy is reported to be useful.

In one patient in our study, pancreatic cancer developed 5 years and 8 months after the biliary diversion operation. Morine et al^4^ reported pancreatic cancer in 14 of 1511 (0.93%) adult patients with PBM which is higher than the incidence rate of pancreatic cancer (around 30 per 100 000) in Japan.^22^ Patients who develop pancreatic cancer after CBD surgery have been reported.^23^ In the present study, fluctuation in serum amylase activity was observed in one patient who developed pancreatic cancer after the operation. Minami et al^24^ reported inflammatory changes in the noncancerous region and dysplasia in the pancreatic duct in a patient with double cancer of the gallbladder and pancreas. Chronic pancreatitis due to PBM may relate to the development of pancreatic cancer, although the relationships between PBM, CBD, or biliary diversion surgery and the development of pancreatic cancer remain unclear due to lack of data. Careful follow‐up for pancreatic cancer is recommended in patients who show fluctuation in serum amylase activity after the biliary diversion operation.

Previous studies have suggested that the proportion of patients with late complications such as cholangitis, hepatolithiasis, and biliary tract cancer after the biliary tract operation seems to be higher in patients with type IV‐A CBD than in those with other types of CBD,^6^, ^9^, ^11^, ^12^ and this study clearly showed that the long‐term outcomes were more unfavorable in patients with type IV‐A CBD compared to those with other types of CBD in terms of late postoperative complications and survival rate. Therefore, in patients with type IV‐A CBD, an exact diagnosis of the dilatation and stenosis of the bile ducts and appropriate treatment is essential to improve outcomes after the biliary diversion operation. Careful long‐term follow‐up with measurement of serum tumor markers such as CA 19‐9 is important after the biliary diversion operation for PBM, especially in patients with IV‐A CBD or repeated cholangitis.

## DISCLOSURE

Conflicts of interest: the authors have no conflicts of interest to disclose.
